# StartReact during gait initiation reveals differential control of muscle activation and inhibition in patients with corticospinal degeneration

**DOI:** 10.1007/s00415-018-9027-0

**Published:** 2018-08-28

**Authors:** Bas J. H. van Lith, Milou J. M. Coppens, Jorik Nonnekes, Bart P. C. van de Warrenburg, Alexander C. Geurts, Vivian Weerdesteyn

**Affiliations:** 1 Department of Rehabilitation, Donders Institute for Brain, Cognition and Behaviour, Radboud University Medical Center, PO Box 9101, 6500 HB Nijmegen, The Netherlands; 2 Department of Neurology, Donders Institute for Brain, Cognition and Behaviour, Radboud University Medical Center, 6500 HB Nijmegen, The Netherlands

**Keywords:** Hereditary spastic paraplegia, Gait initiation, Reticulospinal tract, StartReact effect

## Abstract

Corticospinal lesions cause impairments in voluntary motor control. Recent findings suggest that some degree of voluntary control may be taken over by a compensatory pathway involving the reticulospinal tract. In humans, evidence for this notion mainly comes from StartReact studies. StartReact is the acceleration of reaction times by a startling acoustic stimulus (SAS) simultaneously presented with the imperative stimulus. As previous StartReact studies mainly focused on isolated single-joint movements, the question remains whether the reticulospinal tract can also be utilized for controlling whole-body movements. To investigate reticulospinal control, we applied the StartReact paradigm during gait initiation in 12 healthy controls and 12 patients with ‘pure’ hereditary spastic paraplegia (HSP; i.e., retrograde axonal degeneration of corticospinal tract). Participants performed three consecutive steps in response to an imperative visual stimulus. In 25% of 16 trials a SAS was applied. We determined reaction times of muscle (de)activation, anticipatory postural adjustments (APA) and steps. Without SAS, we observed an overall delay in HSP patients compared to controls. Administration of the SAS accelerated tibialis anterior and rectus femoris onsets in both groups, but more so in HSP patients, resulting in (near-)normal latencies. Soleus offsets were accelerated in controls, but not in HSP patients. The SAS also accelerated APA and step reaction times in both groups, yet these did not normalize in the HSP patients. The reticulospinal tract is able to play a compensatory role in voluntary control of whole-body movements, but seems to lack the capacity to inhibit task-inappropriate muscle activity in patients with corticospinal lesions.

## Introduction

Patients with an upper motor neuron syndrome (UMNS; e.g., stroke, spinal cord injury, cerebral palsy, hereditary spastic paraplegia) have impaired voluntary motor control due to absent or reduced corticospinal output to the alpha motoneurons in the spinal cord. As a result, volitional movements like gait initiation or reaching while standing are impaired in these patients [36, 49, 52]. Such movements are typically preceded by anticipatory postural adjustments (APAs) to optimize postural control during movement [10, 35]. Corticospinal pathways are strongly involved in the control of these APAs [29]. As a consequence, UMNS patients show smaller APA magnitudes and delayed APA latencies compared to healthy controls [13, 25, 36, 49, 52].

Interestingly, recent findings in animals and humans suggest that some degree of voluntary motor control may be taken over by the reticulospinal tract as a compensatory neural pathway [1–3, 17, 19, 41, 55]. In humans, evidence for the potential utility of this compensatory pathway for voluntary movements comes from studies that evaluated the StartReact effect. StartReact refers to the phenomenon that reaction times are greatly accelerated when a startling stimulus is presented simultaneously with an imperative stimulus for executing the requested movement. The exact mechanisms underlying StartReact are, however, still under debate, as the extent of the reaction time acceleration seems to depend on various factors. For instance, the StartReact effect is more likely to occur when there is a high level of motor preparedness and a strong familiarity with the task [24, 32, 33, 38]. Furthermore, the mechanism underlying StartReact appears to depend on the type of action. For example, SAS-induced dexterous hand movements likely engage transcortical pathways, whereas subcortical pathways are more involved in mediating SAS-induced locomotor actions and postural adjustments [24, 32, 33]. Although the exact neural structures that are involved in the StartReact effect are not uncontested, there is ample evidence that StartReact during standing and walking is conveyed by the reticulospinal tract [6, 38, 50, 54].

A previous StartReact study from our group in patients with hereditary spastic paraplegia (HSP) has substantially contributed to the notion of compensation by the reticulospinal tract [41]. In its pure form, HSP is clinically characterized by bilateral muscle spasticity and weakness in the legs, whereas the arms commonly remain unaffected [14, 15, 51]. The main underlying pathological feature in HSP is axonal degeneration of the corticospinal tract [37], particularly affecting the distal parts of the longest descending axons [4]. This degeneration is reflected in lengthened central motor conduction times to the leg muscles upon transcranial magnetic stimulation, e.g., amounting to 150% of reference values from healthy subjects [41]. Indeed, reaction times of voluntary ankle dorsiflexion movements were substantially delayed compared to those of healthy individuals, yet the presentation of a startling acoustic stimulus (SAS) accelerated reaction times to equivalent values in HSP patients and healthy controls [41]. This finding points at an intact reticulospinal system in HSP, and this system may be instrumental for allowing these patients volitional motor control of the lower extremities in the presence of a dysfunctional corticospinal tract.

Previous studies that demonstrated intact StartReact effects on voluntary movements in various groups of patients with UMNS invariably included simple reaction tasks of isolated ankle, hand, wrist or elbow movements performed in a seated position [2, 12, 20, 21, 41]. In contrast, the one study that investigated the StartReact effect in a standing reach task failed to demonstrate a significant SAS-induced acceleration of the requested movement in stroke patients [36]. These discrepant results cast some doubt on the potency of compensatory reticulospinal control for executing complex, multisegmental movements. To shed more light on the potential utility of the reticulospinal system for controlling such movements, we studied the StartReact effect during gait initiation in patients with pure HSP. The APA prior to gait initiation involves concerted tibialis anterior (TA) muscle activation and soleus (SO) inhibition of the stepping leg to move the centre of pressure of the ground reaction forces backwards and towards the stepping leg to accelerate the centre of mass forwards and towards the stance leg [10, 28].

In a gait initiation task in healthy young individuals, it was previously demonstrated that muscle onsets and offsets as well as APA and step onsets were substantially accelerated when a SAS was presented simultaneously with the imperative signal [48]. Based on the majority of StartReact studies in UMNS patients, we hypothesized that HSP patients, compared to healthy controls, would demonstrate delays in all gait initiation parameters when responding to the imperative stimulus alone, but that the presentation of a SAS would result in greater acceleration of muscle onsets and offsets, thus yielding roughly equivalent SAS-induced reaction times in HSP patients and controls.

## Materials and methods

### Ethical approval

The study was approved by the regional medical ethics committee (Commissie Mensgebonden Onderzoek Arnhem-Nijmegen) and was conducted in accordance with the Declaration of Helsinki. All subjects gave written informed consent before the experimental procedures.

### Participants

Twelve patients with autosomal dominant forms of HSP (9 men, 3 women; mean age 51 years, range 27–71 years) and 12 aged-matched healthy controls (9 men, 3 women; mean age 53 years, range 27–71) participated. The patients were recruited from the rehabilitation outpatient clinic of our expert centre for genetic movement disorders. All patients fulfilled the diagnostic clinical criteria for “pure” HSP [51].

### Clinical assessment

Clinical assessments were performed prior to the experiment. Muscle tone of the triceps surae (TS) (ankle dorsiflexion with knee both flexed and extended), TA (ankle plantarflexion), rectus femoris (RF) (knee flexion with hip extended) and biceps femoris (BF) (knee extension with hip flexed) was assessed bilaterally using the Modified Ashworth Scale (0–5), with higher scores indicating more hypertonia [18]. Muscle strength was assessed bilaterally using the Medical Research Council (MRC) scale (0–5) for the TS, TA, RF and BF muscles, with lower scores indicating more muscle weakness [9]. Vibration sense was tested bilaterally at the medial malleolus and at the first metatarsophalangeal joint using the semiquantitative tuning fork (0–8; Rydel Seiffer, Neurologicals, Poulsbo, Washington), with lower scores indicating more sensory loss [45]. We took the mean of the left and right leg for each measure (Table 1).

**Table 1:** Clinical characteristics of HSP patients

	Median	(range)
Rectus femoris		
MAS	1	(1–2)
MRC	4	(3–5)
Biceps femoris		
MAS	1	(0–2)
MRC	4.25	(3–5)
Tibialis anterior		
MAS	0	(0–1)
MRC	4	(3–5)
Triceps surae		
MAS		
Knee extended	1	(0–3)
Knee flexed	1	(0–3)
MRC	4	(3–5)
Forefoot		
Vibration sense	3	(0–6)
Ankle		
Vibration sense	4	(1–6)

All values are means of values for the left and right body side. Vibration sense was tested using a semiquantitative tuning fork (scale range 0–8; Rydel Seiffer, Neurologicals, Poulsbo, Washington). MAS: Modified Ashworth scale (scale range 0–5). MRC: Medical Research Council scale (scale range 0–5)

### Experimental design

#### Familiarization

The subjects received three SASs while standing to familiarize them with the stimuli. The SAS were given through binaural earphones and consisted of 50 ms white noise (1500 Hz) with an intensity of 120 dB (measured by Investigator 2260 and Artificial Ear B&K 6 cc type 4152, Bruel and Kjaer, Nærum, Denmark). The SAS was generated by a custom-made noise generator.

#### Gait initiation

The participants stood in front of a box consisting of two blocks with light-emitting diodes (LED). Illumination of the first LED represented a warning signal and illumination of the second LED represented the imperative stimulus. Warning periods (1–3.5 s) and inter-trial periods (6–10 s) were variable and random. The participants were instructed to stand on the two force plates with their weight equally distributed between the legs. Equal loading of both legs was visually checked online by the primary investigator from the force plate signals. In the case of clear deviation from a symmetrical loading pattern, the subjects were instructed to adjust the loading based on verbal feedback from the primary investigator. As soon as the imperative stimulus was presented, the participants had to start walking as fast as possible and perform at least three steps (one trial), starting with their preferred leg. The preferred leg was defined as the leg with which the participant would kick a football. The participants performed a total of 16 trials; in 25% of these trials a SAS was presented simultaneously with the imperative stimulus using a synchronous analog pulse to the LED box and the startle generator. Prior to the task, the participants performed four practice trials.

### Data collection

Electromyographic (EMG) (ZeroWire, Aurion, Italy) data were collected from both sternocleidomastoid (SCM) muscles and TA, RF and SO muscles of the preferred leg of the participant. The EMG electrodes were placed according to Seniam guidelines [18]. EMG signals (sampled at 2000 Hz) were consecutively band-pass filtered at 20–450 Hz (zero lag, second order Butterworth filter), rectified and low-pass filtered at 30 Hz (zero lag, second order Butterworth filter).

Ground reaction forces under both feet were recorded at a sample rate of 2000 Hz by two force plates (60 × 180 cm each; AMTI Custom 6-axis composite force platform, USA), which were embedded in the surface.

Reflective markers were placed at anatomical landmarks on the heel, ankle and toe of both feet. Marker positions were recorded by an 8-camera 3D motion analysis system (Vicon Motion Systems, United Kingdom) at a sample rate of 100 Hz.

### Data analysis

**Fig. 1:**
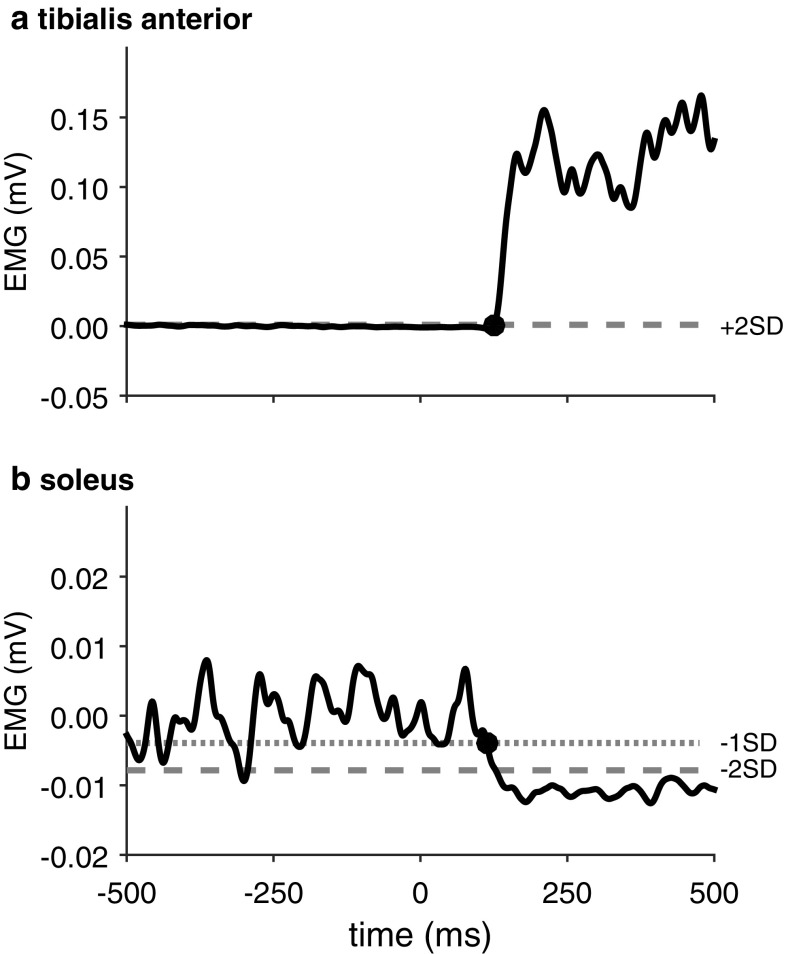
Representative TA and SO EMG signals of the stepping leg in a healthy control participant during gait initiation. TA onset and SO offset are indicated by a dot. Note that the SO baseline is fluctuating much more than the TA baseline. Therefore, TA muscle onset was determined as the instant where the signal exceeded 2 SD above baseline activity, whereas SO muscle offset was determined as the last instant where the EMG signal went below − 1 SD before going below − 2 SD for at least 50 ms

Data analyses were all conducted by the primary investigator. For all signals baseline activities and the respective standard deviations (SD) were calculated over 500 ms prior to the imperative stimulus. The baseline activity was subtracted from all signals. The ensemble average EMG traces of the TA, RF and SO EMG were calculated separately for trials with and without a SAS. We determined muscle onset latencies for TA and RF. We defined the onset as the first instant that a signal exceeded the threshold of 2 SD above baseline activity (Fig. 1a), which was determined by a semi-automatic computer algorithm.

For determining SO offsets, we chose to apply a somewhat more liberal criterion (Fig. 1b). This was done because, compared to the baseline activity of TA and RF, the tonic SO activity at baseline demonstrated greater fluctuations, resulting in large standard deviations. A threshold of -2 SD would therefore have resulted in SO offsets being identified relatively late. We first identified when SO activity went below a threshold of − 2 SD, and then worked backwards to find the instant where the EMG signal exceeded the mean baseline activity − 1 SD. This instant was taken as the SO offset. All onset and offset latencies were visually approved or corrected [39, 41, 42].

For each trial, it was determined whether an anticipatory postural adjustment (APA) occurred prior to the step. To define a weight shift as an APA the force under the stepping leg had to exceed 5% of the total body weight. In addition, the difference between the vertical loading underneath the stepping and stance leg was calculated. The difference had to rise above the threshold of 2 SD above the mean difference 500 ms prior to the imperative stimulus. This moment was defined as the APA onset. In addition, for each APA, the maximum increase in vertical force under the stepping leg was determined and normalized for body weight (BW) [40].

For step onsets, 3D vectors (*x, y* and *z* direction) of the heel and toe markers of the stepping leg were calculated for each trial. The step onset was defined as the first instant when one of the two vectors rose above the threshold of 2 SD above baseline (calculated over 500 ms prior to the imperative stimulus). Step length was determined for each trial separately using the horizontal displacement of heel and toe markers [40].

For each trial with a SAS, we determined whether a startle reflex occurred in SCM. A startle reflex was defined as short latency response in any of the SCM muscles starting within 130 ms following the SAS.

### Statistical analysis

All outcome measures were tested using repeated-measures ANOVA. Group (*HSP patients–healthy controls*) was used as the between-subjects factor and SAS (*SAS–no SAS*) was used as the within-subjects factor. For parameters with interaction effects, post hoc analysis was done to determine the 95% confidence interval (CI) of the mean difference between patients and controls for SAS and no SAS trials separately. Furthermore, we tested for differences in APA occurrences between the two groups using a chi-squared test. We used the chi-squared test also to test for differences in occurrence of startle reflexes between HSP patients and controls in SAS trials.

As a secondary analysis, we tested for differences in onset latencies between SAS trials with a startle reflex (SCM^+^) and SAS trials without a startle reflex (SCM^−^), for those participants who presented both. A repeated-measures ANOVA was used with startle reflex (*SCM*^−^*–SCM*^+^*)* as within-subjects factor and group (*HSP patients–healthy controls*) as between-subjects factor. This secondary analysis was performed because there is an ongoing debate on whether the StartReact effect critically depends on the occurrence of a startle reflex. As such, this analysis was used to determine the potential impact of our decision of analyzing all SAS trials (as opposed to only including SCM + trials) on our primary results and conclusions. Note that for all our other analyses, all SAS trials were included.

Statistical analyses were performed using IBM SPSS Statistics Version 20 for Windows. For all analyses, the *α* level was set at 0.05.

## Results

### EMG onset and offset latencies

**Fig. 2:**
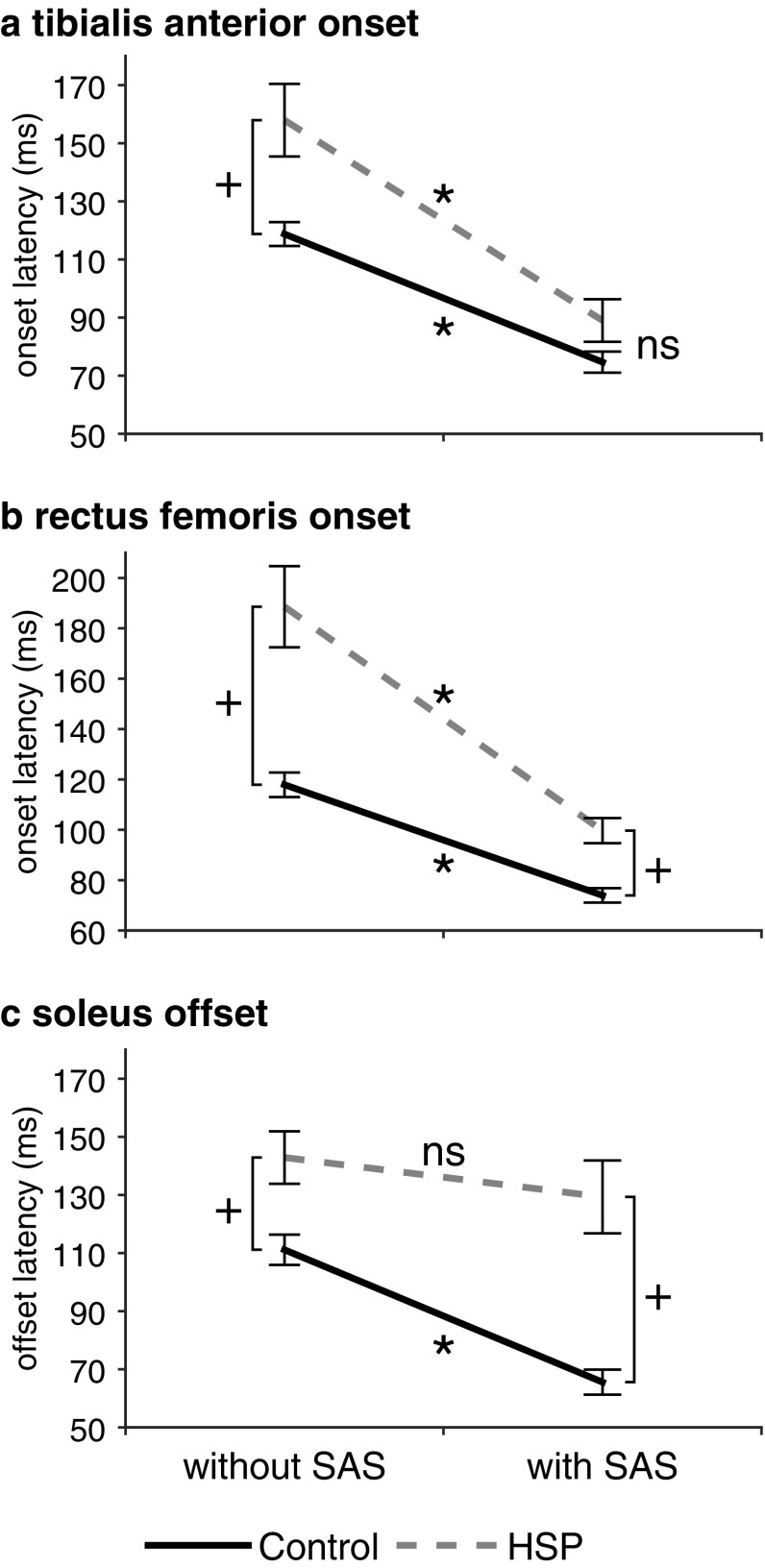
Mean EMG onset/offset latencies (SE) during gait initiation. *Indicates post hoc significant difference between trials with and without a SAS. ^+^Indicates post hoc significant differences between groups with and without a SAS. *ns* not significant

The EMG pattern of the stepping leg during gait initiation was characterized by near-simultaneous TA activation, RF activation and SO inhibition in healthy controls, whereas activation of RF followed shortly after near-simultaneous TA activation and SO inhibition in HSP patients (see Fig. 2).

Without SAS, TA onsets during gait initiation occurred earlier in controls (119 ± 14 ms) than in HSP patients (158 ± 43 ms). Administration of the SAS accelerated these onsets in both groups (*SAS, F*_(1,22)_ = 92.216, *p* < 0.001), as shown in Fig. 2a. Yet, with the addition of the SAS we observed a larger acceleration in TA onsets in the HSP group (89 ± 25 ms) compared to controls (75 ± 13 ms; *SAS* × *group, F*_(1,22)_ = 4.454, *p* = 0.046; *group, F*_(1,22)_ = 8.384, *p* = 0.008). The mean delay in HSP patients was significant without a SAS (95% CI 12–66 ms, *p* = 0.010), but with a SAS the onsets were no longer different from controls (95% CI − 3–31 ms, *p* = 0.093).

With regard to RF onset latencies, the HSP patients showed an overall delay compared to controls (*group, F*_(1,22)_ = 29.254, *p* < 0.001; Fig. 2b). The SAS accelerated RF onsets in both the control group (118 ± 17–74 ± 10 ms) and HSP group (189 ± 56–100 ± 17 ms; *SAS, F*_(1,22)_ = 55.663, *p* < 0.001). Although the SAS-induced acceleration was significantly greater in HSP patients than in the controls (*SAS* × *group, F*_(1,22)_ = 6.388, *p* = 0.019), the delay in RF onsets in the HSP patients compared to healthy controls remained significant with a SAS (mean 26 ms, 95% CI 14–38 ms, *p* < 0.001).

The SO offset without SAS was also delayed in HSP patients (143 ± 31 ms) compared to controls (111 ± 18 ms; *SAS, F*_(1,22)_ = 19.388, *p* < 0.001; Fig. 2c). The SAS accelerated the SO offsets, but in contrast to the results for TA onsets, the SAS-induced acceleration was greater in healthy controls than in HSP patients. Therefore, with addition of the SAS, the SO offsets in healthy controls (66 ± 15 ms) occurred earlier than in HSP patients (129 ± 43 ms; *SAS* × *group, F*_(1,22)_ = 5.687, *p* = 0.026; *group, F*_(1,22)_ = 23.469, *p* < 0.001). Without a SAS, the mean delay in HSP patients was 32 ms (95% CI 10–53 ms, *p* = 0.006), which increased to 64 ms in the SAS trials (95% CI 35–92 ms, *p* < 0.001).

### Anticipatory postural adjustments

**Fig. 3:**
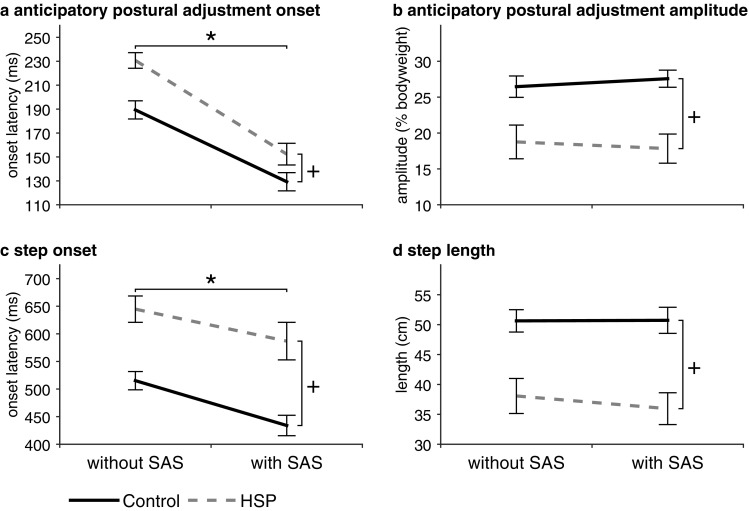
Mean onset latencies (SE) during gait initiation (left graphs) and mean anticipatory postural adjustment amplitudes and step lengths (right graphs). *Indicates significant difference between trials with and without a SAS. ^+^Indicates significant differences between groups with and without a SAS. *ns* not significant

APAs were detected in 87% of the trials in HSP patients, whereas APAs were detected in all the trials of the healthy controls (*χ*^2^_(1,393)_ = 20.855, *p* < 0.001). The HSP patients had delayed APA onsets both without SAS (231 ± 23 ms) and with SAS (152 ± 31 ms) compared to the control group (without SAS: 189 ± 26 ms; with SAS: 129 ± 26 ms; *group, F*_(1,22)_ = 11,62, *p* = 0.003; Fig. 3a). The SAS significantly accelerated APA onsets (*SAS, F*_(1,22)_ = 154.299, *p* < 0.001), yet without differential effects between the two groups (*SAS* × *group, F*_(1,22)_ = 2.693, *p* = 0.115).

APA amplitudes were smaller in HSP patients compared to healthy controls, both without SAS (control: 26 ± 5, HSP: 19 ± 8% BW) and with SAS (control: 27 ± 4, HSP: 18 ± 7% BW; *group: F*_(1,22)_ = 12.355, *p* = 0.002; Fig. 3b). There was no effect of the SAS on APA amplitudes in either group (*SAS, F*_(1,22)_ = 0.016, *p* = 0.901; *SAS* × *group, F*_(1,22)_ = 2.247, *p* = 0.148).

### Step onset and step length

Step onsets in HSP patients were delayed compared to healthy controls (*group, F*_(1,22)_ = 19.898, *p* < 0.001), as shown in Fig. 3c. The SAS accelerated step onsets in both healthy controls (515 ± 57–434 ± 64 ms) and HSP patients (645 ± 83–587 ± 118 ms; *SAS, F*_(1,22)_ = 28.507, *p* < 0.001). There was no differential effect of the SAS between HSP patients and healthy controls (*SAS* × *group, F*_(1,22)_ = 0.801, *p* = 0.381).

No effects of the SAS were found on step length (*SAS, F*_(1,22)_ = 1.168, *p* = 0.291; *SAS* × *groupi F*_(1,22)_ = 1.375, *p* = 0.254; Fig. 3d). In both without SAS and with SAS conditions, HSP patients made shorter steps (38 ± 10 and 36 ± 9 cm) than healthy controls (51 ± 6 and 51 ± 8 cm; *group, F*_(1,22)_ = 16.877, *p* < 0.001).

### Startle reflex

The occurrence of the startle reflex in SCM during SAS trials was 64% in HSP patients and 65% in healthy controls (*χ*^2^_(1,93)_ = 0.000, *p* = 0.989). There were no differences in TA onset between the SCM^−^ trials and SCM^+^ trials in either HSP patients (117 ± 14 and 110 ± 13 ms) or healthy controls (85 ± 4 and 85 ± 4 ms; *SCM, F*_(1,14)_ = 0.715, *p* = 0.412, *SCM* × *group, F*_(1,14)_ = 0.950, *p* = 0.346).

## Discussion

The aim of the present study was to gain more insight about the potency of the reticulospinal tract to act as a compensatory pathway for executing voluntary complex, multisegmental movements. Therefore, we investigated the effects of StartReact on gait initiation in HSP patients. Compared to healthy controls, the HSP group responded to the imperative visual stimulus alone with delayed TA and RF onsets, SO offsets, as well as APA and step onsets. Pairing the imperative stimulus with a SAS resulted in earlier onsets in TA, RF, APA and step onsets both in healthy controls and HSP patients. The SAS-induced acceleration in APA and step onsets was similar between groups, whereas a significantly greater StartReact effect was observed in TA and RF onset latencies in the HSP patients than in the controls, resulting in (near)-normal TA and RF onsets in the HSP group. In response to the visual stimulus alone, we observed TA onsets and SO offsets at approximately the same time. In the healthy controls, the SAS similarly accelerated TA onsets and SO offsets, such that the relative timing between these events was not affected. Yet, remarkably, no SAS-induced acceleration in SO offsets was observed in the HSP patients.

Our study adds to the existing body of knowledge on StartReact effects in patients with upper motor neuron lesions [20, 21, 31, 36, 41] by demonstrating that patients with HSP showed greatly accelerated reaction times in a gait initiation task, as an example of a common voluntary whole-body movement. Without a SAS, there was a difference in TA onset between HSP patients and healthy controls, probably due to a delayed corticospinal conduction time. With the SAS, the normalization of TA onsets in HSP patients suggests that these patients now used the same neural pathway as healthy controls (i.e., the reticulospinal tract) to generate the SAS-induced movements, irrespective of whether acceleration of reaction times may have been limited by physiological floor effects. It is important to mention that the observed SAS-induced reaction times in this study (75–89 ms) are in the same order of magnitude as those previously reported during both voluntary ankle dorsiflexion and gait initiation in various populations [40, 41, 48]. In addition, our SAS-induced TA onsets are in line with previously reported startle reflex onsets in TA [5, 41], which further supports the notion that the SAS-induced response is conveyed by the reticulospinal tract.

This finding complements previous work on single-joint movements. In our previous study in HSP patients, delayed onset latencies of TA activity and ankle dorsiflexion movements were observed in a single-joint reaction task, yet with a SAS the patients’ reaction times were comparable to those of healthy individuals [41]. Similarly, in stroke survivors, onset latencies of isolated elbow flexion and hand extension movements were delayed without a SAS, whereas these reaction times were also normalized with the SAS [20, 21]. To our knowledge, only one previous study has investigated the StartReact effect in a voluntary whole-body movement in a group of UMNS patients. During forward standing reaches with the paretic arm, people with stroke demonstrated delayed onsets of both the anticipatory postural adjustment (APA) and the focal reaching movement compared to healthy control subjects. Administration of a SAS led to a significant reduction in reaction times in the controls. In contrast, in the people with stroke the SAS did not speed up APA onsets, whereas it even caused a further delay in reaching onsets [36]. The discrepancy between these and our present findings may be related to damage of cortical areas responsible for motor preparation (e.g., pre-motor cortex, supplementary motor cortex) after stroke, whereas HSP (in its pure form) does not affect neurons originating from these secondary motor areas. As the StartReact phenomenon typically depends on the requested movement being readily prepared when the SAS is administered, (partial) sparing of these cortical motor preparation areas seems imperative, with the degree of sparing likely becoming more critical as the task becomes more complex.

In this study, we found no difference in SAS-induced onset latencies between SCM+ and SCM- trials, which is consistent with previous StartReact studies that included lower-extremity movements [33, 38, 39, 41]. In contrast, small but significant differences have been demonstrated in several studies that focused on upper-extremity movements [8, 21, 34]. Based on these results, it was previously suggested that a true StartReact effect could only occur when a startle reflex in SCM was also elicited. Yet, at present, startle reflexes and acceleration of motor responses by a startling stimulus are considered to be dissociated phenomena [38]. The small difference between SCM+ and SCM- trials that has been reported by some authors is likely explained by the presence of a startle reflex in SCM being a marker of preparedness, with a higher level of preparedness in SCM+ trials leading to shorter reaction times [32, 33, 38]. Although it remains elusive why this effect is not observed in lower-extremity movements, our results confirm that the presence of a startle reflex is not conditional for the occurrence of the StartReact effect.

An unexpected finding of the present study was the lack of acceleration of soleus inhibition in the HSP patients upon administration of the SAS. In contrast, the SAS did accelerate SO offsets in the healthy controls, which is in agreement with the observations from a previous study that investigated the StartReact phenomenon in a gait initiation task in healthy young participants [48]. In that study, SO offsets and TA onsets during gait initiation were both accelerated to latencies of ~ 50–70 ms by a SAS [7, 48], which is consistent with the latencies observed in our healthy control group. StartReact effects on muscle inhibition have also been demonstrated in healthy young participants in a reaction time paradigm where they had to actively inhibit a baseline elbow flexion force with and without a SAS [7]. Hence, it appears that our HSP patients differ from healthy participants in their lack of SAS-induced inhibitory motor control. As both SO inhibition and TA activation are known to contribute to the generation of the APA [10], the observed disparity in excitatory and inhibitory StartReact effects in the HSP patients may also explain why their APA onsets were not accelerated to healthy control levels with the SAS.

The results of the current study raise the question which mechanism(s) may underlie the absent StartReact effect on muscle inhibition in the HSP patients. Here we can only speculate, as our study was not designed to address this unexpected finding. One suggestion may be that inhibitory control of the reticulospinal tract on spinal motoneurons is less potent than its excitatory effects. This relative difference between inhibitory and excitatory strength would be in line with previous observations in cats, where it has been shown that the reticulospinal tract has fast-conducting excitatory (activating) fibers that project directly onto motoneurons, while inhibitory reticulospinal projections merely appeared to be indirect (i.e., via interneurons) [47]. Yet, under this assumption, one would expect SAS-induced differences in relative timing of TA onsets and SO offsets in the healthy controls as well, which we did not observe.

Another possibility is that HSP differentially affects the dorsal and medial fascicles of the reticulospinal tract. Animal studies have suggested that inhibitory commands are predominantly conducted by the dorsal reticulospinal tract (which runs closely to the corticospinal tract [11]), whereas the medial reticulospinal tract mainly conducts excitatory commands from the reticular formation [30, 53]. Hence, the lack of SAS-induced acceleration in SO offsets may be due to affliction of the dorsal reticulospinal tract, while the medial reticulospinal may remain unaffected in the presently studied genotypes of HSP. Yet, we are unaware of any (post-mortem) studies in patients with HSP in support of this hypothesis.

A third and perhaps most plausible mechanism is that the inhibitory SAS-induced command may lack strength to overcome the tonic calf muscle activity that is present when standing upright. Indeed, calf muscle tone is typically higher in UMNS patients compared to healthy controls. Due to the lack of descending inputs in UMNS, muscle activity can cause motoneurons to activate voltage-dependent persistent inward currents. These persistent inward currents can lead to self-sustained firing of motoneurons, resulting in a long-lasting involuntary enhancement of muscle activity [16] that may override the effect of the inhibitory SAS-induced reticulospinal command. However, there is no direct evidence in support of this suggestion and further research is warranted to elucidate the mechanisms underlying defective StartReact effects on muscle inhibition in HSP.

The present findings may shed new light on the functional utility of the reticulospinal tract for bypassing defective corticospinal control. Animal studies have provided strong evidence for the potential of a compensatory role of the reticulospinal tract in recovery of upper-extremity motor function (for review: see Baker [1]) and lately also of lower-extremity function. For instance, recovery of function after complete corticospinal lesions was shown to coincide with an increased output from the reticulospinal system as measured with intracellular recordings [3, 17, 23, 55]. Also in humans after stroke, the notion of reticulospinal contributions to functional recovery is gaining support, particularly those concerned with severe damage to the primary motor cortex and/or the corticospinal tract [1, 22, 26, 27, 44]. However, reticulospinal motor control has limitations compared to corticospinal control due to the greater dispersion of reticulospinal projections on spinal motor neurons [46], limiting the degree of refined fractionated movements. The results of the present study suggest that reticulospinal motor control may also be inferior because this system lacks the capacity to inhibit task-inappropriate muscle activity. Yet, the exact mechanisms remain elusive and can only be speculated upon. Together, these considerations may explain why in HSP patients spasticity and lack of refined motor control are often more prominent impairments than muscle weakness [43]. During our task of gait initiation, the functional disadvantages of defective soleus inhibition on, for instance, step onset appeared to be rather minimal; however, poor inhibitory control may be more detrimental to performance in other postural tasks, such as postural perturbations. Further research is needed to fully understand the potential and limitations of compensatory reticulospinal motor control in upper motor neuron syndrome.
